# Guidance for defining outcomes in clinical trials

**DOI:** 10.1371/journal.pmed.1005275

**Published:** 2026-10-21

**Authors:** Xiangji Ying, Tianjing Li, Joanne E. McKenzie, Matthew J. Page, Kiran Ninan, Jean-Pierre Oberste, Colby J. Vorland, Andrew W. Brown, Riaz Qureshi, Nicholas J. DeVito, Ian J. Saldanha, Martin Offringa, Nancy J. Butcher, Sirui Zhang, Phi-Yen Nguyen, Joe Menke, Veronica Ka Wai Lai, Halil Kilicoglu, Evan Mayo-Wilson

**Affiliations:** 1 Department of Epidemiology, University of North Carolina Gillings School of Global Public Health, Chapel Hill, North Carolina, United States of America; 2 Department of Ophthalmology, University of Colorado Anschutz Medical Campus, Aurora, Colorado, United States of America; 3 Methods in Evidence Synthesis Unit, School of Public Health and Preventive Medicine, Monash University, Melbourne, Victoria, Australia; 4 Department of Epidemiology and Biostatistics, Indiana University School of Public Health-Bloomington, Bloomington, Indiana, United States of America; 5 Department of Biostatistics, University of Arkansas for Medical Sciences, Little Rock, Arkansas, United States of America; 6 Arkansas Children’s Research Institute, Little Rock, Arkansas, United States of America; 7 Nuffield Department of Primary Care Health Sciences, University of Oxford, Oxford, United Kingdom; 8 Department of Epidemiology, Johns Hopkins Bloomberg School of Public Health, Baltimore, Maryland, United States of America; 9 Child Health Evaluative Sciences, The Hospital for Sick Children Research Institute, Toronto, Ontario, Canada; 10 Department of Psychiatry, University of Toronto, Toronto, Ontario, Canada; 11 Department of Epidemiology, School of Public Health, Brown University, Providence, Rhode Island, United States of America; 12 School of Information Sciences, University of Illinois Urbana-Champaign, Champaign, Illinois, United States of America

## Abstract

**Background:**

Defining outcomes completely before conducting clinical trials helps to mitigate concerns about reporting biases; however, there is limited guidance to help investigators define outcomes completely.

**Methods:**

Building on a previous framework, we developed guidance for defining trial outcomes completely and consistently. We reviewed literature, developed preliminary guidance, and refined guidance iteratively through pilot testing. We randomly selected 225 randomized controlled trials (RCTs) from ClinicalTrials.gov, which evaluated 3,424 outcomes. Two raters followed a structured approach to define each outcome. When raters encountered outcomes they could not define, we refined our guidance. We continued this process until no further changes were needed. We finalized the guidance through multiple drafts and discussions among authors.

**Findings:**

In clinical trials, a completely defined outcome includes six elements: outcome domain, specific measurement, specific metric, cutoff (if applicable), variable type, and time point. We developed guidance for naming specific measurements for both subjective and objective outcomes. We expanded on prior work by describing more comprehensive categories for specific metrics. To be consistent with the estimand framework, we replaced “method of aggregation” with two distinct elements that describe values in individuals (rather than summary measures): “variable type” and “cutoff.” We further defined “cutoff” with three sub-elements, and we defined “variable type” as whether the value for each individual is continuous or categorical.

**Conclusions:**

Trialists and sponsors could use this framework and guidance to define outcomes in trial registrations, protocols, and results reports. When all six elements are defined, readers can better determine whether reported outcomes were prespecified and whether different trials assessed the same outcomes.

## Author summary

**Why was this study done?:**

- Clinical trials measure outcomes related to concepts such as pain, blood pressure, or survival to estimate whether interventions help or harm patients.
- If an outcome is not defined completely before a trial begins, researchers can later analyze results in multiple ways and select the outcome definition that makes the treatment look best.
- There is little practical guidance on how to define an outcome completely to prevent reporting biases.

**What did the researchers do and find?:**

- Building on an earlier framework published in 2011, we identified six pieces of information needed to define a trial outcome completely: the construct of interest, data collection instruments, method for assigning values to individuals, any threshold used to group people, what type of value it is, and the time of assessment.
- The guidance has since been used in two further studies of several thousand outcomes, with good agreement between independent raters and between human raters and an automated system.

**What do these findings mean?:**

- Defining outcomes using these elements brings trial reporting closer to international standards for stating what a trial is designed to estimate.
- The guidance could help researchers and trial sponsors describe outcomes completely and consistently in trial registrations, protocols, and published results.
- Complete outcome definitions alone will not prevent all forms of biased reporting; better tools and systems are also needed to help researchers prepare registrations and protocols.

## Abstract 3

In this Guidelines and Guidance article, Xiangji Ying and colleagues provide updated guidance—including a six-element framework with worked examples—for defining outcomes completely and consistently in clinical trials.

## Introduction

Registering clinical trials and defining outcomes completely can help mitigate reporting biases. That is, different outcome definitions can lead to different numerical results. By pre-specifying outcome definitions, investigators reassure readers that reported results are consistent with planned outcomes and that results were not selected based on their magnitude or statistical significance. Laws and policies [1–3], funders [4,5], and journals [6–8] require that clinical trialists register trial outcomes prospectively and report results completely [9]. Notably, the International Committee of Medical Journal Editors (ICMJE) requires that trials be registered “to prevent selective publication and selective reporting of research outcomes” [8].

Registering clinical trials might reduce the risk of two related reporting biases. First, registration enables readers to evaluate whether prespecified outcomes are reported or omitted in subsequent reports (e.g., journal articles reporting results). Second, registration allows users to evaluate whether outcomes are reported using the same or different definitions compared with planned outcome definitions; for example, using the same measurement instruments and time points as planned. In practice, current trial registration requirements might mitigate the first type of bias (non-reporting of outcomes) while failing to address selective reporting related to changes in outcome definitions [10–12].

In addition to the risk of reporting biases, incomplete outcome definitions contribute to two related problems. First, incomplete outcomes might be difficult to compare across different sources for the same trial (e.g., comparing a study protocol with a final report). Second, results might not be combined and interpreted correctly across trials (e.g., in a systematic review) when it is unclear whether trials assessed the same outcomes [13].

In 2011, Zarin and colleagues proposed a structured framework to define outcomes on ClinicalTrials.gov [14]. They argued that an outcome definition comprises five elements: (1) an outcome domain (e.g., pain); (2) specific measurement (e.g., McGill Pain Questionnaire); (3) specific metric (e.g., change from baseline); (4) method of aggregation (e.g., mean, proportion); and (5) time point (e.g., 12 weeks) [14]. Their framework was incorporated in regulations governing trial reporting. It was also incorporated in the quality-control review criteria for ClinicalTrials.gov [15], which have also evolved over time. Their framework was also adopted by reporting guidelines, and it is reflected in both CONSORT 2025 [16,17] and SPIRIT 2025 [18,19].

Sponsors and investigators might struggle to register outcomes completely because there is limited operational guidance to help apply the framework by Zarin and colleagues. For example, the framework [14] and subsequent guidelines define “specific measurement” as the name of a tool or instrument used to assess an outcome, but not all outcomes require a tool or instrument. Zarin and colleagues [14] and the SPIRIT [18–20] and CONSORT [16,17,21] reporting guidelines provide examples of how to define each element, but their examples are not sufficiently comprehensive or generalizable to register all outcomes completely. For instance, most previous authors have not explained how to register composite outcomes or outcomes that involve multiple criteria (e.g., health problems defined using multiple instruments at multiple times).

There are many examples of outcomes that have been registered on ClinicalTrials.gov and other registries that might appear to be defined completely but omit one or more elements [22]. For example, registrations commonly define outcomes by naming only a measure, such as "Patient Health Questionnaire-9 (PHQ-9)”, and time points such as “weeks 0, 4, 8, and 12" (Fig 1). Because one measure might be compatible with many distinct outcome definitions (e.g., up to 27 results might be compatible with the registered outcome definition for PHQ-9), incomplete registrations might not effectively limit opportunities for selective reporting.

**Fig 1:**
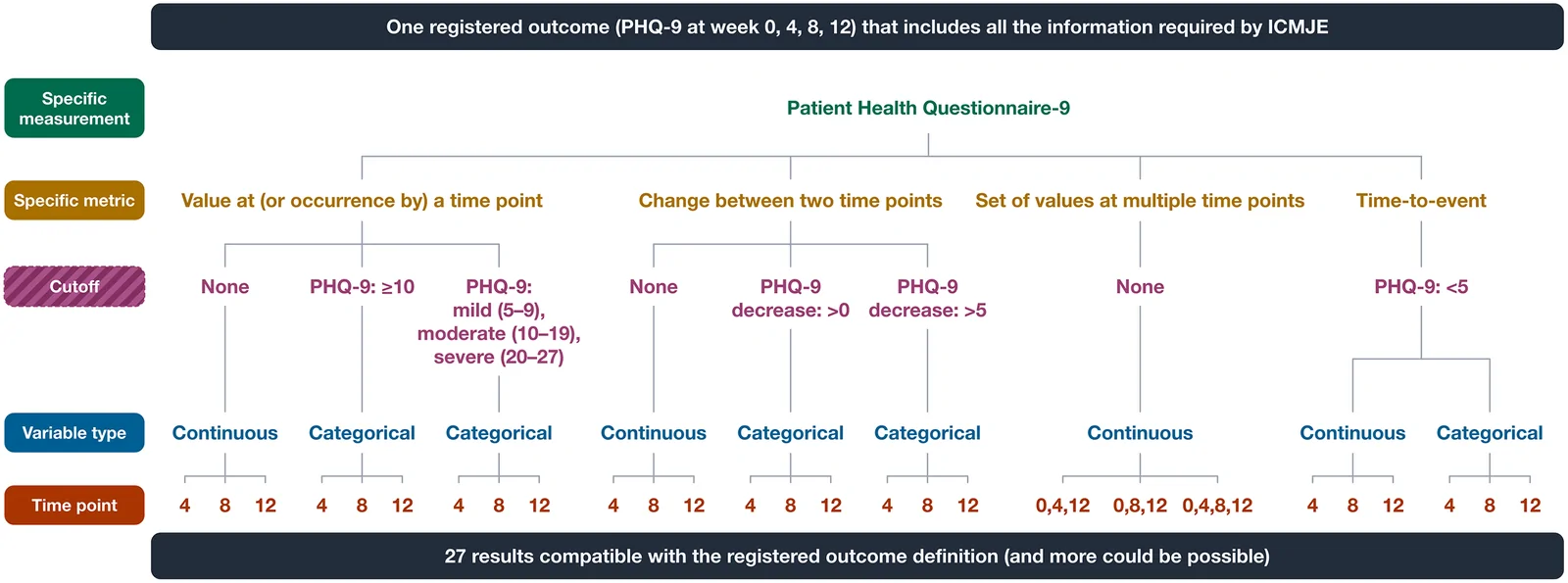
An example of a prospectively registered but incompletely defined outcome. This example outcome (PHQ-9 at week 0, 4, 8, and 12) meets current ICMJE registration requirements but could be consistent with dozens of results because it is defined incompletely.

Since 2011, guidelines for clinical trials and other studies of interventions have adopted the “estimand” framework. An estimand is what a study aims to estimate [23–25], and the estimand framework defines an outcome as “the variable (or endpoint) to be obtained for each patient” [23]. In addition to an outcome, an estimand also includes the population and interventions of interest, handling of intercurrent events, and summary measures for aggregating outcomes within groups and comparing them between groups. Although not yet adopted in the ICMJE trial registration policy, other contemporary sources such as the International Council for Harmonisation E9(R1) specify that estimands should be defined in trial protocols, statistical analysis plans, and reports [16–19,23]. Following the estimand framework, we update the framework by Zarin and colleagues to distinguish outcome from its summary measure, and we provide operational guidance to help sponsors and investigators define clinical trial outcomes completely. This guidance should help trialists implement recommendations in SPIRIT, CONSORT, and other guidelines for registering and reporting RCTs.

## Methods

Guidance for defining outcomes was developed through a two-stage process [26]. First, we developed preliminary guidance using frameworks from previous publications [14,17–21,27]. Second, we refined that guidance through pilot testing and revision.

In the second stage, two raters (from XY, KN, JPO, EMW, CJV, SZ, AWB, RQ, NJD, and MJP) independently and in duplicate applied our draft guidance using a random sample of randomized controlled trials (RCTs) registered on ClinicalTrials.gov. We began pilot testing with 50 trials (S1 Fig). Then we added additional trials in batches, refining our guidance whenever an outcome could not be defined using our rules. We continued this process until successive batches produced no new cases requiring modifications. We stopped pilot testing and revision after 225 trials.

We used the development dataset to develop an automated system that identifies whether outcomes on ClinicalTrials.gov are defined completely. Subsequently, we manually rated the completeness of outcome definitions in 150 trials (2,292 outcomes) registered on ClinicalTrials.gov and used those ratings to validate the automated system [10]. We also applied the framework to evaluate 210 trials (2,543 outcomes) published in ICMJE member journals in 2025 [11]. In both empirical studies, we found good to excellent interrater agreement among human raters and between humans and the automated system.

Structured registry data were used to develop and pilot test the guidance. Specifically, we reviewed four data fields for prospectively registered outcomes, and we reviewed 12 data fields for outcomes with results (S1 Text). We restricted our sample to phase III or “Not Applicable” phase RCTs, which might be expected to define outcomes completely when they calculate sample sizes. On ClinicalTrials.gov, the “Not Applicable” phase is used for device trials and for interventions not regulated by FDA (e.g., behavioral interventions). We did not restrict by registration date. We restricted the sample to trials that were registered before the actual or estimated study start date (i.e., prospectively registered) and had results available on ClinicalTrials.gov at the time of our search (January 4, 2024). This allowed us to examine two registration versions for each trial, which included prospectively registered outcomes and outcomes with reported results, respectively. We also required each trial to have uploaded a study protocol and statistical analysis plan in case we might examine them in a future study.

Guidance to define outcomes was shared with all co-authors, who include experts in epidemiology and biostatistics, clinical trials, reporting guidelines, reporting biases, evidence synthesis, and computational tools for assessing biomedical texts. The co-authors provided feedback over multiple drafts and attended two 2-hour meetings to resolve outstanding questions about rules to define outcomes, terminology, and presentation.

## A six-element framework to define outcomes

An outcome in a clinical trial is the value, or set of values, for each individual participant (or other unit of observation) that will be used to calculate the estimand [23–25]. A completely defined outcome includes six elements (Fig 2). Within this framework, we use the term “outcome” rather than “endpoint”, which is sometimes used interchangeably [16–21,23–25,28]. While the meaning of “endpoint” is appropriate and intuitive for time-to-event outcomes because participants are censored after specified events (i.e., follow-up *ends*), the term “outcome” does not imply a terminal event, so it is appropriate for both time-to-event data and for data recorded at multiple times [29].

**Fig 2:**
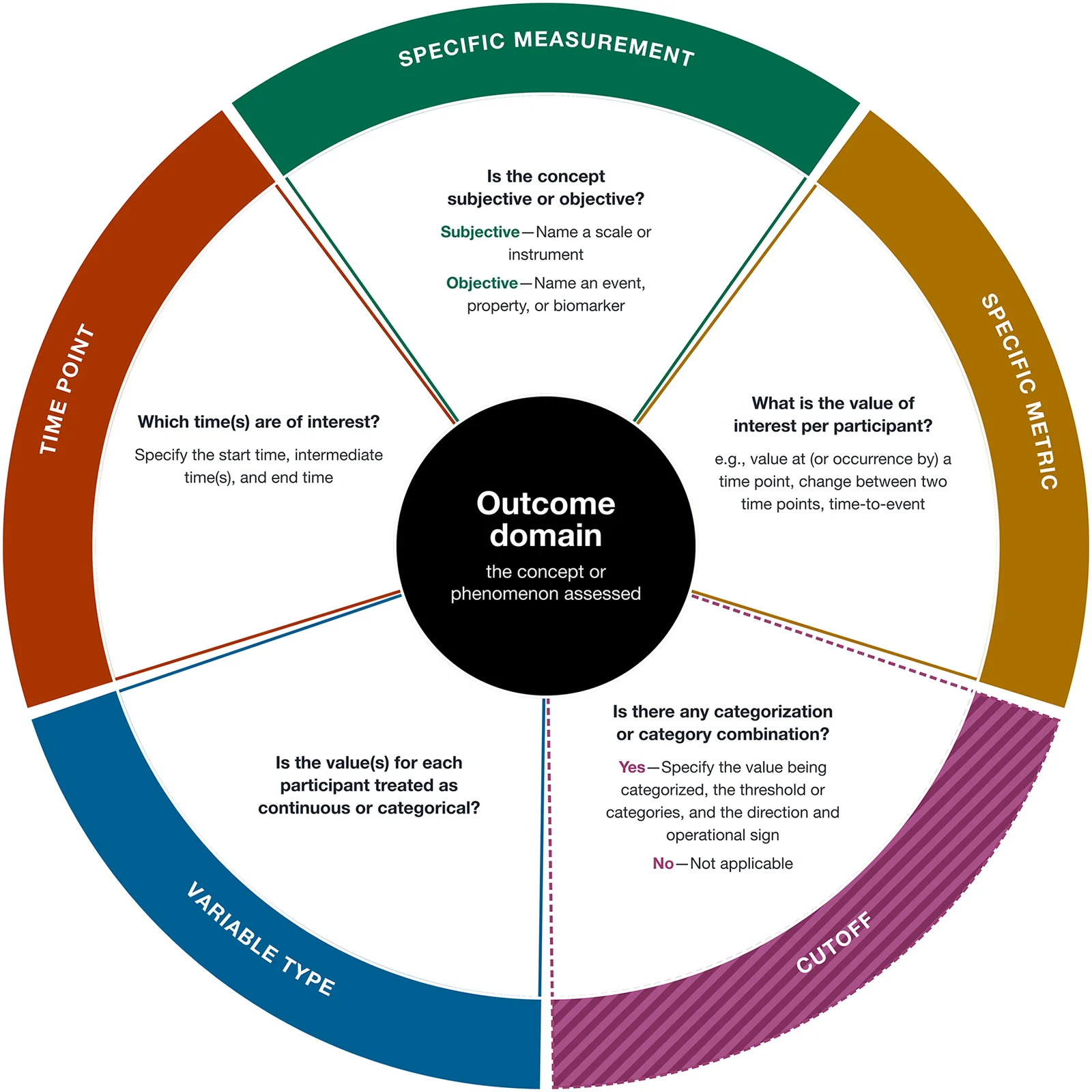
The six-element framework for defining outcomes in clinical trials.

Four elements from the Zarin and colleagues framework [14] align with current guidance regarding outcomes in the estimand framework, so we operationalize and explain them without substantial modifications. We modify one element, “method of aggregation”, to reflect the distinction between an *outcome* and the *summary measure* in the estimand framework (S1 Table). That is, we retain the individual participant-level content of what Zarin and colleagues called “method of aggregation,” but we exclude summarization across participants, which relates to a different part of an estimand. We also break “method of aggregation” into two elements, *variable type* and *cutoff,* to which Zarin and colleagues alluded in their examples [14].

In an individually randomized trial, *variable type* refers to whether the individual participant-level value is continuous or categorical. It is not the *summary measure* (a different part of an estimand), but it does constrain which summary measures might be used. For example, each participant’s score on the modified Rankin Scale (0–6), which measures degree of disability and dependence in daily activities, is an ordinal category that could be treated as either continuous or categorical. Potential summary measures depend on whether each participant’s outcome is defined as a continuous or categorical variable.

We separate *cutoff* from *variable type* because cutoffs are necessary to define some outcomes and because the use of a cutoff does not necessarily make an outcome categorical, which some previous articles assume. For example, time spent above or below a threshold, or time until a threshold is crossed, are *continuous* outcomes defined by cutoffs. As explained subsequently, we treat cutoff as a standalone element to encourage transparent reporting when cutoffs are applied, not to encourage dichotomization or categorization.

Consistent with the estimand framework and with Zarin and colleagues [14], we do not consider outcome priority (i.e., whether an outcome is primary, secondary, or other) to be part of an outcome definition. Priority characterizes the role or importance of an outcome (or estimand) rather than how its value is derived. Consistent with these frameworks and with reporting guidelines such as SPIRIT [18–20] and CONSORT [16,17,21], we also distinguish outcome definitions from data collection methods, which might include the person who collected data (e.g., trained nurse, participant self-report), methods for capturing data (e.g., routinely collected data, mobile devices), and quality control (e.g., duplicate measurement, rest period before measurement).

In the following sections, we illustrate how each element should be defined in documents such as protocols and registrations, and we include decision trees and worked examples. S1 Appendix provides further examples of incompletely defined outcomes, each paired with two complete versions: one defined using our framework, which does not include information about the summary measure, and one that also meets the ClinicalTrials.gov requirement to specify a "method of aggregation.”

### Element 1: Outcome domain

An *outcome domain* is the concept or phenomenon assessed [14,16–21,30]. For example, the Core Outcome Measures for Effectiveness Trials (COMET) initiative developed a high-level taxonomy for outcome domains in medical research [31]. Outcome domains might be defined in more or less granularity.

Identifying an outcome domain can help trialists select appropriate instruments to measure concepts and phenomena of interest. For example, trialists might identify a concept such as “drug adherence” and then consider various measures (e.g., pill count, blood concentration) along with their measurement properties (e.g., validity, reliability, and responsiveness), clinical utility, implications for trial conduct, and costs.

When outcome domains are not stated explicitly, inferring them can be challenging. For example, “drug blood concentration” could be used to assess “drug adherence” or “pharmacokinetics”, depending on context. Although trial registers do not include specific fields for outcome domains, researchers can include this information in free text fields. Researchers might also explain outcome domains in study protocols and statistical analysis plans—which might be published separately or attached to registrations—to help readers understand the intended purpose, rationale, and interpretation of outcomes [20,21].

### Element 2: Specific measurement

A *specific measurement* is the name of a scale or instrument used to assess a concept, or the name of a phenomenon that can be observed directly (Fig 3). Trialists sometimes use multiple instruments to assess a single outcome domain, so pre-specifying specific measurements can limit opportunities for reporting bias.

**Fig 3:**
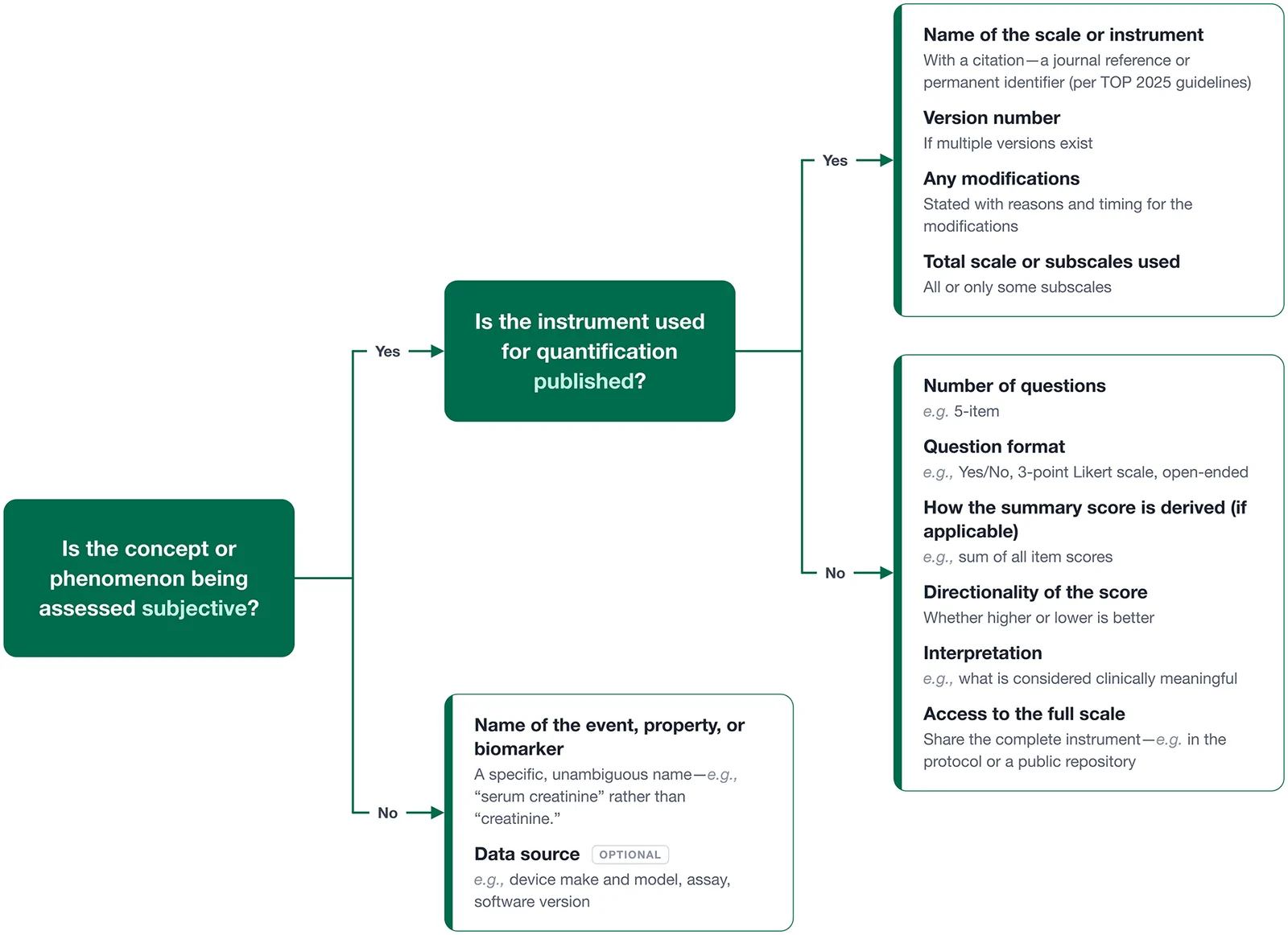
Guidance for defining specific measurements.

To define a specific measurement, investigators should first consider whether the concept or phenomenon being assessed is subjective or objective. Subjective characteristics often require a scale or instrument for quantification. For example, quality of life is subjective and is typically measured using named instruments (e.g., SF-36 [32], EQ-5D [33], and EORTC QLQ-C30 [34]). A complete outcome definition would name the instrument, including the version number (if there are multiple versions), any modifications (ideally with rationale), and whether the total or subscales (i.e., all or some subscales) will be used. Some instruments (e.g., the Brief Pain Inventory [BPI]) contain two or more subscales and do not yield a single overall score. In such cases, the subscale name should be specified (see Box 1). To facilitate clinical interpretation, trial registrations and protocols might also describe instruments’ properties and details of how they operate.

**Box 1. Example 1: Brief pain inventory.**

**Background**

- Trialists are interested in measuring pain-severity at 4 weeks.
- Pain-severity is a subjective concept that cannot be observed directly and must be quantified with an instrument.
- The trialists plan to use the Brief Pain Inventory (BPI), which comprises two subscale scores—pain-severity and pain-interference—and does not yield a single overall score.
- They find many trials have registered outcomes such as “BPI at 4 weeks.”

**Questions to consider**

- Which subscales will be used?
- If both, do they carry the same priority (e.g., both primary) or different priorities (e.g., one primary, one secondary)?
- Is the value of interest the score at 4 weeks, or is it the score at baseline minus the score at 4 weeks?
- Will the score be treated as a continuous value or categorized using a cutoff?

**A completely defined outcome**

The trialists decide they are interested in the pain-severity subscale, analyzed as a continuous score at the 4-week visit, and define the elements as follows:

- Outcome domain: pain-severity.
- Specific measurement: BPI, pain-severity subscale.
- Specific metric: value at (or occurrence by) a time point.
- Cutoff: not applicable.
- Variable type: continuous.
- Time point: 4 weeks.

They state the outcome compactly as “pain-severity measured using the BPI pain-severity score (continuous) at 4 weeks.”

When custom forms or unpublished instruments are used to measure subjective characteristics, they should be made available following best practices for sharing research materials [35]. Notably, ClinicalTrials.gov and other registers allow investigators to attach files. In the structured fields of a trial registration, a complete definition of a specific measurement would include: the number of questions (e.g., “1-question,” “5-question”), the question format (e.g., Yes/No, 3-point Likert scale, rating scale, and open-ended), directionality (higher is better or higher is worse), and the names of the concepts or phenomena being assessed. When using custom questionnaires, explaining how they will be used and interpreted (e.g., possible score range, procedure for calculating the total score) might also mitigate concerns about multiple testing and selective nonreporting of results.

Some objective phenomena can be assessed without a scale or instrument. For example, mortality and days-in-hospital might be recorded through direct observation or records. In such cases, the specific measurement is the precise name of the event being observed (e.g., cancer-specific mortality, all-cause mortality). Trial registrations and protocols might also specify the corresponding data source, which is part of the data collection methods.

Other objective phenomena, including some physical properties and biomarkers, can be assessed using instruments that might be considered interchangeable in research. For example, scales to measure body weight at different sites of a multi-center trial might differ in model and make, but weighing scales are typically regarded as equivalent. Similarly, fasting blood glucose might be measured using a glucometer or via standard laboratory testing; differences between these approaches are often treated as random measurement error. Because it might be uncommon for investigators to measure the same biomarker using multiple instruments per participant (e.g., multiple glucometers per person), not specifying this information is unlikely to introduce opportunities for reporting biases in many cases. Moreover, a single instrument can sometimes measure multiple quantities (e.g., a sphygmomanometer can measure both systolic and diastolic blood pressure), so the name of an instrument might be insufficient to define the phenomenon of interest.

For objective phenomena measured by interchangeable instruments, the specific measurement often refers to a property or biomarker, not the instrument used. Where applicable, the property or biomarker being assessed should be named precisely. For example, “fasting blood glucose” is more precise than “blood glucose.” Broad terms that can be assessed in various ways do not constitute a specific measurement. For instance, “glucoregulation” might be described as an “outcome domain” rather than a specific measurement. Similarly, “anti-pneumococcal antibody levels” is imprecise while “serum IgG antibody against pneumococcal serotype 3 polysaccharide” is precise because it defines the sample type (i.e., serum rather than blood or plasma) and the analyte (i.e., IgG antibody rather than IgM antibody).

In some cases, trialists might also include details about the instruments used to assess physical properties and biomarkers (e.g., device make and model, software version). For example, “visual field,” which describes how wide a person can see, is a common outcome domain in vision research. It is typically measured using perimetry, which involves a combination of hardware, visual stimuli, testing patterns, and algorithms. In a registration, trialists might specify that they plan to calculate a “visual field index.” In study protocols and other documents that can be attached to structured registrations, trialists might provide other details of the visual field assessment that would be needed for replication in clinical practice or research.

### Element 3: Specific metric

A *specific metric* is how the participant-level quantity or quantities of interest are obtained from a participant's assessments at one or more time points (Fig 4; S2 Table). Depending on the estimand of interest, the outcome for each individual participant might be a single value (e.g., needed to estimate a value at the end of a study) or a set of values (e.g., needed to estimate rate of change).

**Fig 4:**
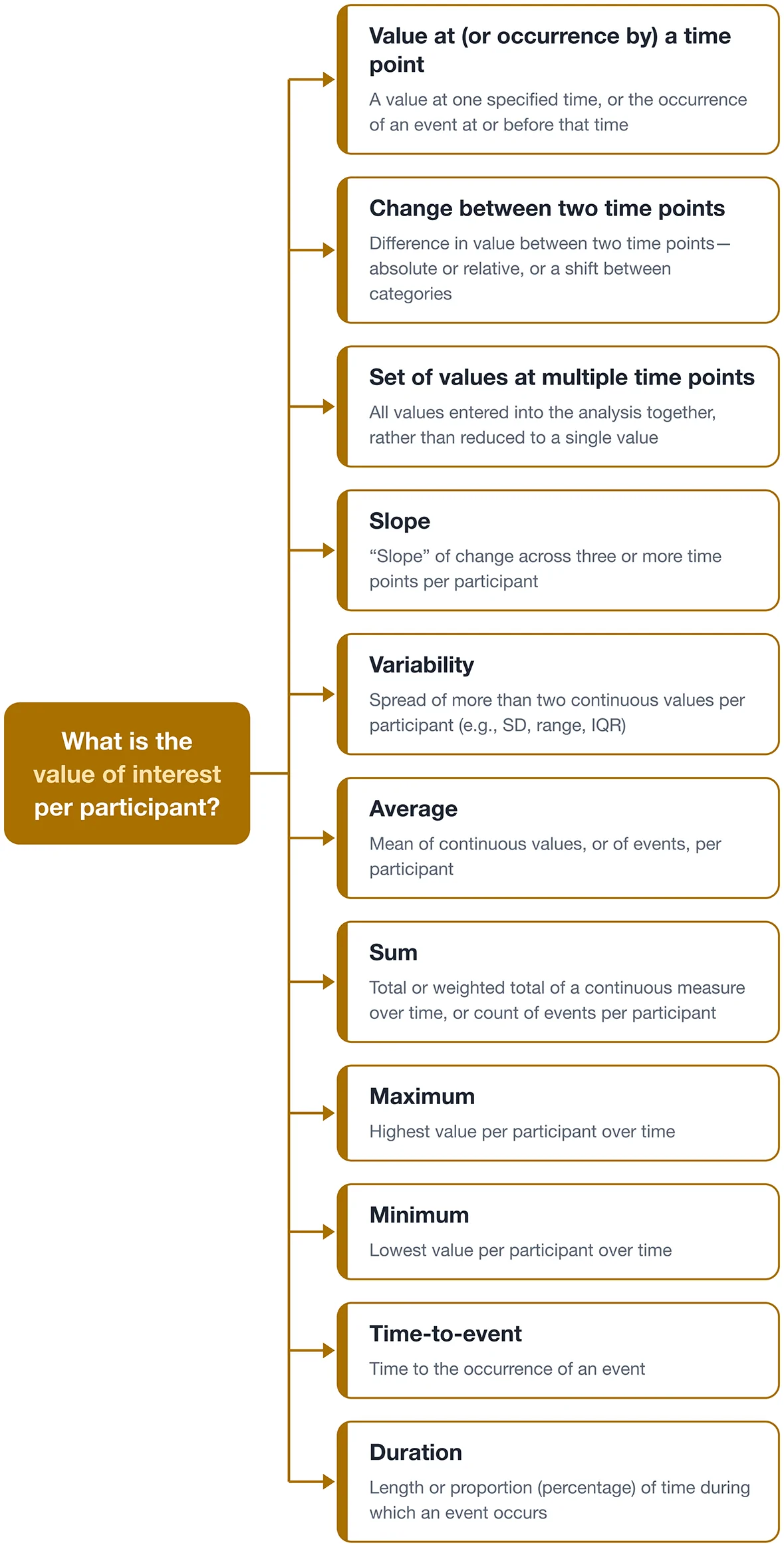
Guidance for defining specific metrics. The figure presents common examples of specific metrics identified in our review of thousands of outcomes; uncommon metrics might not be represented. For further details and examples of each metric type, see S2 Table.

For interval, ratio, or ordinal measurements, the specific metric might be the value at a time point (e.g., weight at the end of the study), the difference in values between two times (e.g., difference between weight at the end of the study and weight at baseline), or the set of values at multiple time points (e.g., weight at 3, 6, and 12 months entering a mixed-effects model together). Some continuous measures are monitored repeatedly in each participant (e.g., blood glucose) and the specific metric might be a summary of those measurements in each participant, such as the mean (e.g., Mean Amplitude of Glycemic Excursions [MAGE] per participant), variability, sum, minimum, maximum, or the slope (e.g., individual participant’s annualized slope of eGFR).

For nominal measurements (e.g., categories or discrete events), including continuous scales that are categorized, the specific metric might be the value at or occurrence by a time point or a change in category. Investigators should beware that naming an event might not be sufficient to define the specific metric; for example, “death” is ambiguous because the event might be treated as binary status at a particular time (i.e., value at or occurrence by a time point) or the survival time of each participant (i.e., time to event). For events that can occur multiple times in an individual, the specific metric might be defined as a binary status (e.g., “one or more falls by the end of the study”), a count (e.g., “number of falls per person”), the average number of occurrences per person per unit of time (e.g., “number of drinks per week”), or a series of binary statuses at multiple time points (e.g., whether one or more falls occurred in each of three consecutive months) (see Box 2).

**Box 2. Example 2: Adverse events.**

**Background**

- Investigators of a drug trial are interested in measuring potential harms.
- They find many trials have registered outcomes such as “adverse events” or “rate of adverse events.”

**Questions to consider**

- Which specific adverse events would be measured and how would they be assessed?
- Is the outcome a composite measure of “any adverse event” or individual adverse events?
- Would several prespecified adverse events be collected systematically using the same assessments across all participants in all groups, or would events be collected nonsystematically through spontaneous reports by participants at any time point?
- Is the value of interest the binary status (i.e., whether each participant had one or more events), the number of events per participant, or the frequency of events occurring in each participant?

**A completely defined outcome**

The investigators decide they are interested in any adverse events up to 1 year. They specify the six elements as:

- Outcome domain: harm.
- Specific measurement: any adverse events. (Data collection details: data on adverse events will be collected by asking participants about unwanted effects at monthly visits, through spontaneous reports by participants and providers at any time point, and through review of electronic medical records and death records.)
- Specific metric: value at (or occurrence by) a time point.
- Cutoff: not applicable.
- Variable type: categorical.
- Time point: 1-year (follow-up horizon).

They state the outcome compactly as “Harms, assessed as whether each participant experienced one or more adverse events within 1-year. Data on adverse events will be collected by asking participants about unwanted effects at monthly visits, through spontaneous reports by participants and providers at any time point, and through review of electronic medical records and death records.”

Specific metrics related to person-time might be defined as (1) *time* *before* an event or (2) *duration* *of* an event. The former is often relevant for events that can occur once in a person (or once for a participant in a trial). For events that can recur in people, trialists might assess duration until the first event, duration until a subsequent event, or duration since the last event. After categorization, continuous measures might be assessed using time (e.g., time to lose 5% of initial body weight). For events or activities that are monitored repeatedly or continuously, trialists might analyze the length or proportion of time during which events occur.

### Element 4: Cutoff (if applicable)

A *cutoff* is a rule for categorizing a continuous or ordinal value (e.g., grouping HbA1c into <6%, 6% to 7%, and >7% to classify glycemic control), or for reducing or combining the levels of a categorical variable (e.g., classifying adverse events as severe or worse using NCI Common Terminology Criteria for Adverse Events (CTCAE) Version 4.0 grade ≥3) (Fig 5). Here, we exclude values described solely for interpretive purposes (e.g., to explain what is considered a high or low value) or for defining subgroups based on baseline characteristics, which are not part of outcome definitions.

**Fig 5:**
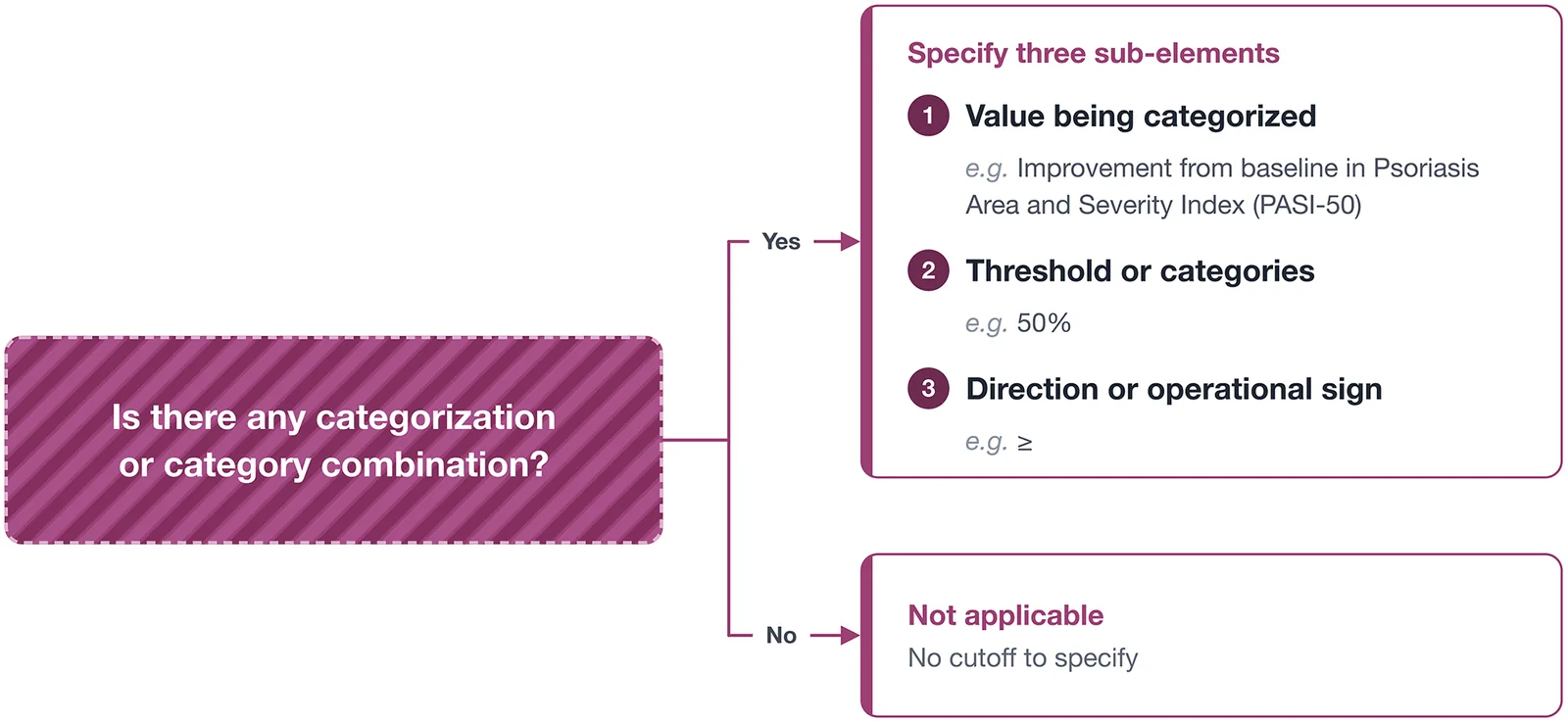
Guidance for defining cutoffs.

We include *cutoff* as an element because cutoffs are necessary to define many outcomes and because choosing cutoffs after examining data can contribute to risk of bias. Cutoffs are not needed to define all outcomes, and dichotomizing or categorizing a variable that could be analyzed continuously is often undesirable because it discards information and can reduce statistical power [36].

A completely defined cutoff includes three sub-elements (Fig 5; Box 3). First, the value in each individual, which is defined by the specific measurement and sometimes the specific metric. Second, the specific threshold or categories of interest. That is, values on continuous or ordinal measures might be dichotomized using a single threshold or categorized using ranges, and values on categorical scales might be collapsed for analysis. Third, the direction (e.g., increase, decrease) and operations (e.g., =, >, <, ≥, ≤) used.

**Box 3. Example 3: Achieved target blood pressure.**

**Background**

- Investigators are interested in measuring blood pressure.
- They find many trials have registered outcomes such as “achieved blood pressure at 1-year”.

**Questions to consider**

- How will “target blood pressure” be defined? Will both systolic and diastolic blood pressure be measured and used to define the target?
- What will be the cutoff imposed for one or both of the blood pressure measurements?
- Is the value of interest the binary status (achieved or not) at a particular time or time to achieve the target level?

**Two completely defined outcomes**

The investigators decide they are interested in whether and when participants reach the target during the first year, and they define one primary outcome and one secondary outcome.

First, they define the six elements for the primary outcome as:

- Outcome domain: blood pressure.
- Specific measurement: systolic and diastolic blood pressure.
- Specific metric: time to event.
- Cutoff: Systolic blood pressure (SBP) <120 mm Hg AND diastolic blood pressure (DBP) <80 mm Hg.
- Variable type: continuous.
- Time point: from randomization to 1 year (assessments at 3, 6, 9, 12 months)

They state the primary outcome compactly as “Time to achieve target blood pressure (SBP < 120 mm Hg AND DBP < 80 mm Hg) from randomization to 1-year (assessments at 3, 6, 9, and 12 months).”

Second, they define the six elements for the secondary outcome as:

- Outcome domain: blood pressure.
- Specific measurement: systolic and diastolic blood pressure.
- Specific metric: value at (or occurrence by) a time point.
- Cutoff: Systolic blood pressure <120 mm Hg AND diastolic blood pressure <80 mm Hg.
- Variable type: categorical.
- Time point: 1 year (assessments at 3, 6, 9, and 12 months)

They state the secondary outcome compactly as “Whether the participant’s blood pressure was within the target range (SBP < 120 mm Hg AND DBP < 80 mm Hg) at 1-year follow-up.”

### Element 5: Variable type

The *variable type* describes whether the value for each individual is treated as continuous or categorical (Fig 6). It describes the nature of the individual participant-level value that enters the aggregate-level analysis, which might differ from the form in which the data were originally collected. For example, pain might be recorded on a 0–10 numeric rating scale, but the value entering analysis might be a binary indicator of pain reduction ≥2-point.

**Fig 6:**
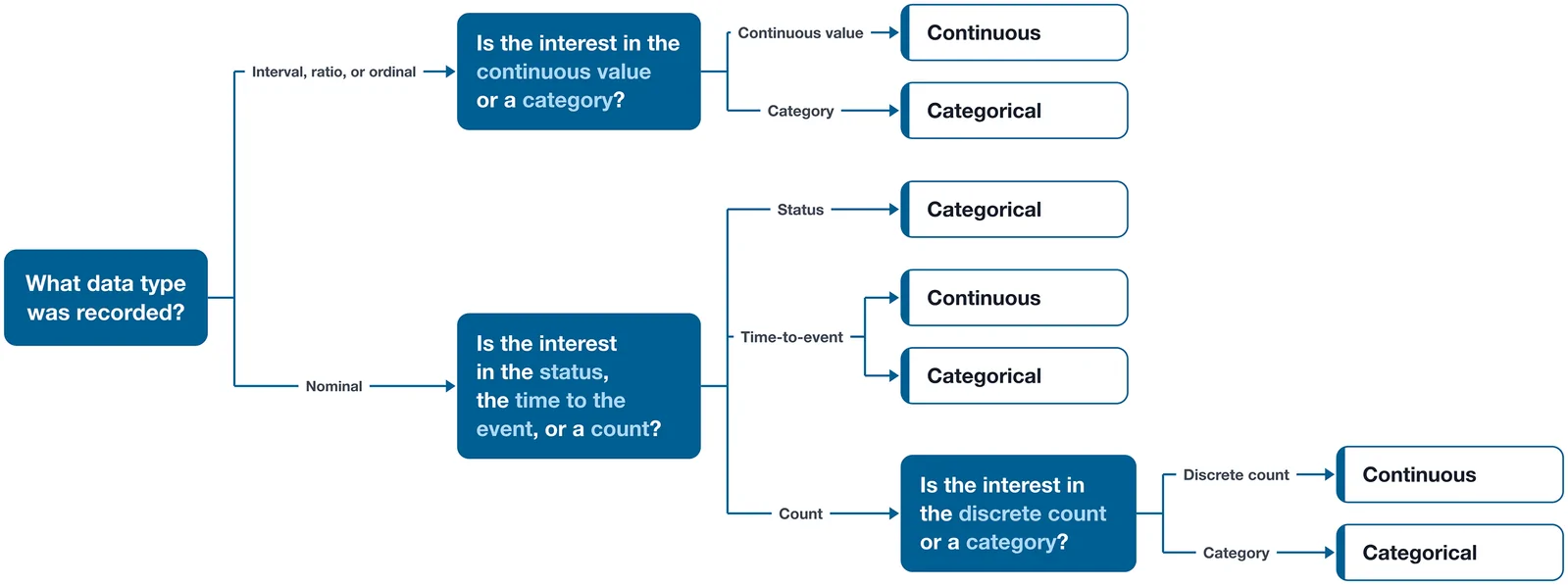
Guidance for defining variable type. Time-to-event data might be analyzed both continuously (e.g., median survival) and categorically (e.g., survival rate).

In some cases, variable type follows from decisions recorded in other elements. On one hand, applying cutoffs to continuous measurements often yields categorical values. On the other hand, an ordinal score might be treated as a numeric score or as ordered categories with no cutoff. A time-to-event metric could be treated as a continuous value (i.e., continuous time to the event) or a categorical value (i.e., whether the event occurred by a specified time). Stating the variable type eliminates any ambiguity.

Broadly, a value is continuous when it is a quantity on a scale (e.g., an interval or ratio measure) or a count; it is categorical when each participant is classified into one of a set of levels (e.g., a nominal measure).

Although variable types are distinct from summary measures used to aggregate information across individuals, variable types can sometimes be inferred from summary measures. For example, means and medians are used to summarize continuous values. Proportions and odds are used to summarize categorical values. Investigators might name a variable type in a trial registration (e.g., “change in BMI (kg/m^2^) from baseline to 1-month (continuous outcome)”), but they more commonly name a summary measure that implies a variable type (e.g., “mean change in BMI (kg/m^2^) from baseline to 1-month”) (see Box 4). The latter approach adheres to the ClinicalTrials.gov guidance to specify the *method of aggregation*.

**Box 4. Example 4: Body mass index.**

**Background**

- Investigators of a weight-loss trial are interested in measuring body mass index (BMI) at baseline, 8 weeks, and 6 months.
- BMI is always derived from height and weight, so its components need not be named—"BMI" alone is a specific measurement.
- They find many trials have registered outcomes such as "BMI at baseline, 8 weeks, and 6 months."

**Questions to consider**

- Will BMI be treated as a continuous value or as a category? Both are common.
- If categorical, which cutoffs are of interest?
- How will the three time points be used? For example, do the investigators aim to estimate BMI at each time point separately, estimate the change in BMI from baseline at each post-baseline time point, or are they interested in the rate at which BMI changes?

**A completely defined outcome**

The investigators decide they are interested in each participant's BMI as a categorical value at 8 weeks and at 6 months, with BMI category at 8-weeks being the primary outcome and BMI category at 6-months as a secondary outcome. They specify the six elements for the primary outcome as:

- Outcome domain: adiposity.
- Specific measurement: BMI.
- Specific metric: value at (or occurrence by) a time point.
- Cutoff: <18.5, 18.5 ≤ BMI < 25.0, 25.0 ≤ BMI < 30.0, ≥30.0.
- Variable type: categorical.
- Time point: 8 weeks.

They state the primary outcome compactly as "Adiposity measured using Body Mass Index category (<18.5, 18.5 ≤ BMI < 25.0, 25.0 ≤ BMI < 30.0, ≥30.0) at 8-weeks."

### Element 6: Time point

A *time point* is a moment when an assessment will be recorded (Fig 7; S3 Table). Depending on the estimand of interest, outcomes might be defined by one or more time points. For example, investigators might plan to use one assessment per participant to estimate the difference between two groups at the end of a study. Alternatively, investigators might plan to use two assessments per participant to estimate the difference in change between two groups from baseline (T0) to the end of a study (T1). To estimate the difference in the rate of change between groups, investigators might plan to use three or more assessments per participant (e.g., T0, T1, T2, and T3).

**Fig 7:**
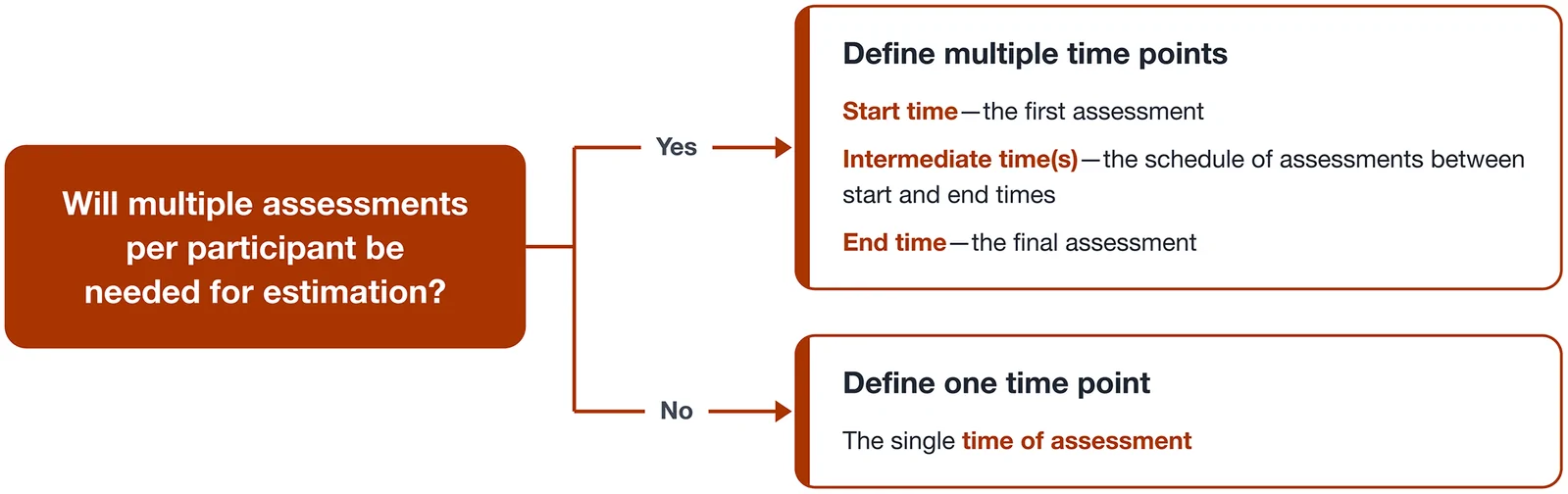
Guidance for defining time points.

In a clinical trial, investigators might also conduct assessments at multiple time points for purposes other than estimating intervention effects (e.g., to support handling of missing data). These assessments should be distinguished from time points that define outcomes. Moreover, values recorded at baseline might be used to define some outcomes (e.g., “change from baseline”), but variables measured at baseline only are not “outcomes” because the effects of interventions must occur after randomization.

Many estimands focus on treatment effects at a single time point. In study protocols and statistical analysis plans, it is often clear that investigators plan to use one measure to estimate effects at separate time points; in registrations, entering each time point as a unique outcome might be clearer than including multiple time points in one field (Box 4). Similarly, when investigators plan to give different priority to different time points (e.g., 12-month “primary,” 1- and 6-month assessments “secondary”), registering them as separate outcomes might help avoid ambiguity and reduce risk of perceived outcome “switching” (e.g., *post ho*c changes in priority).

When investigators aim to estimate something that requires values to be collected at multiple time points (e.g., in repeated-measures or time-to-event analyses), those time points all contribute to one outcome. In those cases, a complete outcome definition would include either (1) all the time points or (2) the start time, end time, and intermediate times (e.g., every 3 months after randomization for 3 years, continuously over 24 hours) (Box 3; Box 5). In practice, intermediate times are sometimes left unspecified. Because the same estimand can be estimated from different schedules—for instance, the difference in mean rate of change over 12 weeks could be estimated from weekly assessments through week 12 or from assessments at baseline, week 8, and week 12—investigators should state prospectively when values will be collected. Doing so reduces the risk that only a subset of the collected time points would be included in the analysis because they support the desired conclusion, which is a form of selective reporting bias. For events that can be observed only at certain times, such as change in tumor size, the frequency of assessment should be described.

**Box 5. Example 5: Progression-free survival.**

**Background**

- In a cancer trial, investigators are interested in progression-free survival at 5-years.
- Progression-free survival is a composite time-to-event measure, and the component events and the rules for detecting progression can be defined in multiple ways.

**Questions to consider**

- What counts as an event? Some studies count progression or death from any cause, while other studies count progression or cancer-specific death.
- Studies classify progression differently. Which criteria will be used (e.g., Response Evaluation Criteria in Solid Tumors [RECIST])?
- What is the participant-level value of interest? Whether a participant survives 5 years or each participant's time to progression or death?
- Although a time point (year 5) is named, progression can be detected only at scheduled assessments. What is the assessment schedule?

**A completely defined outcome**

The investigators decide they are interested in each participant's time from randomization to progression or death from any cause, over 5 years, with progression assessed every 3 months, specifying the six elements as:

- Outcome domain: cancer survival.
- Specific measurement: cancer progression or death from any cause; progression is assessed by RECIST 1.1 and defined as at least a 20% increase in the sum of target-lesion diameters relative to the smallest sum recorded on study, with an absolute increase of at least 5 mm; the appearance of one or more new lesions; or unequivocal progression of nontarget lesions.
- Specific metric: time-to-event.
- Cutoff: none at the outcome level; one for the measurement component, as reported under specific measurement.
- Variable type: continuous.
- Time point: from randomization through 5 years; progression assessed every 3 months, death ascertained continuously.

They state the outcome compactly as "Time to progression or death from any cause, through year 5, with progression assessed every 3 months by RECIST 1.1 and defined as at least a 20% increase in the sum of target-lesion diameters relative to the smallest sum recorded on study, with an absolute increase of at least 5 mm; the appearance of one or more new lesions; or unequivocal progression of nontarget lesions."

### Additional considerations for composite and other complex outcomes

Composite and other complex outcomes are not defined by a single event, scale, or property. A composite outcome occurs if one or more of several component events occur (i.e., components connected with “or”). A multi-criteria outcome is defined by meeting a set (or parts of a set) of criteria simultaneously (i.e., components connected with “and”). Other complex outcomes are derived by combining two or more measurements using algorithms. For these outcomes, each event, scale, or property might have a corresponding specific metric, time point, and cutoff (if applicable) (Fig 8; Box 5).

**Fig 8:**
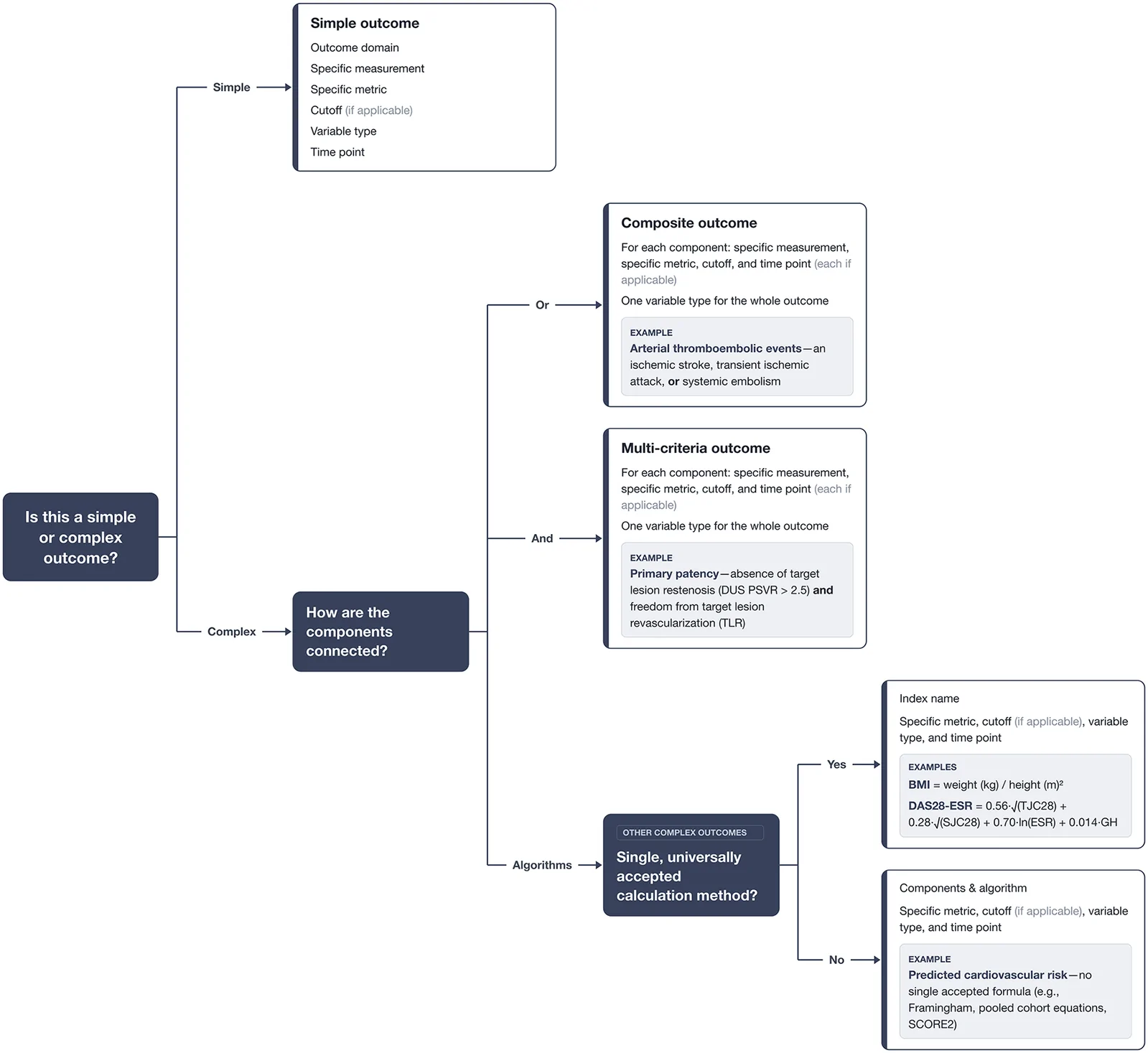
Guidance for defining complex outcomes. BMI, body mass index; DAS28-ESR, Disease Activity Score in 28 joints using the erythrocyte sedimentation rate; TJC28 / SJC28 = tender / swollen joint count (28 joints); ESR, erythrocyte sedimentation rate; GH, patient global health (visual analog scale); DUS PSVR, duplex ultrasound peak systolic velocity ratio; SCORE2 = Systematic COronary Risk Evaluation 2.

### Implications and limitations

This updated framework and guidance could help sponsors and investigators define outcomes in registries, study protocols, and statistical analysis plans. Complete definitions would support adherence to registration and results submission requirements [15,37–39], the ICMJE trial registration policy [8], and CONSORT/SPIRIT reporting guidelines [16–21]. Complete definitions would also help investigators adhere to policies and guidelines, including the US Final Rule for Clinical Trials Registration and Results Information Submission (42 CFR Part 11) [2], which requires responsible parties of applicable clinical trials to submit prespecified outcome information to ClinicalTrials.gov. Explicit criteria and categories to define outcomes could facilitate further development of automated methods to assess outcome definitions and changes in outcome definitions [10].

Like Zarin and colleagues [14], we focused on outcomes in clinical trials. To pilot test this framework, we sampled trials randomly, most of which were individually randomized. Similar approaches based on the Zarin and colleagues framework have been used to assess outcomes in observational studies [12,40]. Nonetheless, minor adaptations and different examples might be needed to address other trial designs and study types. For example, outcomes for clusters—in which values are aggregated across individuals to form one outcome for the cluster, which then contributes to a summary measure across clusters—might need to be defined somewhat differently compared with outcomes in individuals.

Although we interpret “method of aggregation” as related to summary measures rather than outcomes in the estimand framework, we agree with Zarin and colleagues that defining summary measures and other components of estimands is essential to limit reporting biases. Many laws and policies written 10–20 years ago do not require that study protocols and statistical analysis plans be shared before beginning enrollment in clinical trials [2,8,41,42]. By comparison, psychology and other disciplines adopted the practice of "preregistration" after biomedicine adopted the practice of “registration.” Some disciplines understand "preregistration" to include a structured registration and also the public deposition of a study protocol and statistical analysis plan containing substantially more than the minimum information required by registers such as ClinicalTrials.gov [43]. Merely defining outcomes in registers is insufficient to prevent selective publication and selective reporting of research results [11], and trust in clinical trials could be improved by adopting more stringent norms that have emerged in other disciplines [35,44].

This guidance, and reporting guidelines such as SPIRIT, might help motivated investigators write better registrations and protocols. ICMJE and other organizations could strengthen their registration policies to require that outcomes (and potentially other components of estimands) be defined completely before enrolling participants [8]. Some funders already make continued payments contingent on open science practices, and funder policies could be strengthened to require that outcomes and estimands be defined completely before studies begin enrollment. It might be unreasonable (and ineffective) to expect that all clinical trialists become experts in all aspects of trial regulation, registration, and reporting. Instead, academic medical centers, drug manufacturers, and other sponsors could ensure they have adequate staff who are trained to support investigators in these tasks [45]. Although most organizations that operate trial registers cannot compel investigators to adhere to best practices—and registries are sometimes compelled by laws and policies to post records they consider problematic—several simple changes could promote better registrations. For example, current guidance sometimes focuses on compliance with laws and policies that do not require trialists to register all elements prospectively (S4 Table). To help trialists enter information accurately and completely, registries could offer more comprehensive guidance, introduce structured fields to define each element of an outcome (and other components of estimands), and allow categorical responses where applicable. For example, trialists already enter structured information when posting results on ClinicalTrials.gov; allowing trialists to use those menus to define outcomes prospectively would provide additional opportunities to reduce concerns about whether planned outcomes and reported outcomes match.

## Supporting information

S1 Fig Sampling flowchart. Flowchart showing the sampling and selection of studies used to develop the framework.
(TIFF)

S1 Text Fields on ClinicalTrials.gov we reviewed to define outcomes and results. (DOCX)

S1 Appendix Examples of incompletely defined and completely defined outcomes. (DOCX)

S1 Table Frameworks for defining outcomes. A comparison of past and current frameworks for defining outcomes.
(DOCX)

S2 Table Common metrics describing values for individual participants in clinical trials. (DOCX)

S3 Table Do's and don'ts when defining time points (adapted from ClinicalTrials.gov). (DOCX)

S4 Table Outcome fields of 18 WHO recognized primary registers. (DOCX)

## Abbreviations

BMI: body mass index
BPI: Brief Pain Inventory
COMET: Core Outcome Measures for Effectiveness Trials
CTCAE: Common Terminology Criteria for Adverse Events
DBP: diastolic blood pressure
ICMJE: International Committee of Medical Journal Editors
MAGE: Mean Amplitude of Glycemic Excursions
RCTs: randomized controlled trials
RECIST: response evaluation criteria in solid tumors
SBP: Systolic blood pressure.
