# Supplementary material for: Guidance for defining outcomes in clinical trials
Source: PLoS Med. 2026 Oct 21;23(10):e1005275. doi: 10.1371/journal.pmed.1005275 (PMC13719018; doi:10.1371/journal.pmed.1005275)
Supplement: Supplementary file 1 [file pmed.1005275.s002.docx]

## **S1 Text. Fields on ClinicalTrials.gov we reviewed to define outcomes and results**

*From Outcome Measures module (for prospective registrations)*

1. Outcome Measure Type [Select one: Primary, Secondary, Other]
2. Outcome Measure Title [Free text]
3. Outcome Measure Description [Free text]
4. Outcome Measure Time Frame [Free text]

*From Outcome Measure and Statistical Analysis module (for reported results records)*

1. Outcome Measure Type [Select one: Primary, Secondary, Other Pre-specified, Post-Hoc]
2. Outcome Measure Title [Free text]
3. Outcome Measure Description [Free text]
4. Outcome Measure Time Frame [Free text]
5. Measure Type [Select one: Count of Participants, Mean, Median, Least Squares Mean (LSM), Geometric Mean, Geometric LSM, Number, Count of Units]
6. Measure of Dispersion/Precision [Select one: Not Applicable, Standard Deviation, Standard Error, Inter-Quartile Range, Full Range, _____ % Confidence Interval, Geometric Coefficient of Variation]
7. Row/Category Title [Free text]
8. Overall Number of Participants/Units Analyzed [Numeric value]
9. Number of Participants/Units Analyzed [Numeric value]
10. Unit of Measure [Free text]
11. Method for the Statistical Test of Hypothesis [Select one: ANCOVA, ANOVA, Chi-Squared, Chi-Squared (Corrected), Cochran-Mantel-Haenszel, Fisher Exact, Kruskal-Wallis, Log Rank, Mantel Haenszel, McNemar, Mixed Models Analysis, Regression (Cox), Regression (Linear), Regression (Logistic), Sign Test, t-Test (1-Sided), t-Test (2-Sided), Wilcoxon (Mann-Whitney), Other (_____)]
12. Estimation Parameter [Select one: Cox Proportional Hazard, Hazard Ratio (HR), Hazard Ratio (Log), Mean Difference (Final Values), Mean Difference (Net), Median Difference (Final Values), Median Difference (Net), Odds Ratio (OR), Odds Ratio (Log), Risk Difference (RD), Risk Ratio (RR), Risk Ratio (Log), Slope, Other (_____)]
