# Supplementary material for: Guidance for defining outcomes in clinical trials
Source: PLoS Med. 2026 Oct 21;23(10):e1005275. doi: 10.1371/journal.pmed.1005275 (PMC13719018; doi:10.1371/journal.pmed.1005275)
Supplement: Supplementary file 2 [file pmed.1005275.s003.docx]

## S1 Appendix. Examples of incompletely defined and completely defined outcomes

The incompletely defined outcomes in this appendix are drawn from ClinicalTrials.gov. Each incompletely defined outcome is presented as the outcome measure with a time frame in brackets. For each, we provide two completely defined examples: one following our framework and one written to meet ClinicalTrials.gov quality-control review criteria. The ClinicalTrials.gov quality-control criteria require the method of aggregation (summary measure) to be specified. Although the summary measure is not part of an outcome definition, terms such as "proportion" or "mean difference" often imply a metric and/or variable type. The two complete examples do not necessarily describe the same outcome, and outcome domain is omitted because registries have no field for it.

| Example 1 | Specific measurement | Specific metric | Cutoff | Variable type | Time point |
| --- | --- | --- | --- | --- | --- |
| *Incomplete* | | | | | |
| Pain intensity [Day 1 after surgery] | Not defined: pain intensity cannot be observed directly and requires a scale or instrument for quantification | Not defined | Not applicable | Not defined | Day 1 after surgery |
| *Complete* | | | | | |
| Continuous Visual Analog Scale (VAS) score at day 1 after surgery | Visual Analog Scale (VAS) | Value at (or occurrence by) a time point | Not applicable | Continuous | Day 1 after surgery |
| Mean Faces Pain Scale – Revised (FPS-R) [At Day 1 after surgery] | Faces Pain Scale – Revised (FPS-R) | Value at (or occurrence by) a time point | Not applicable | Continuous | Day 1 after surgery |

| Example 2 | Specific measurement | Specific metric | Cutoff | Variable type | Time point |
| --- | --- | --- | --- | --- | --- |
| *Incomplete* | | | | | |
| 17-item Hamilton Depression Rating Scale (HAM-D17) [Baseline, 4-weeks] | 17-item Hamilton Depression Rating Scale (HAM-D17) | Not defined: “Baseline” alone cannot be an outcome in a clinical trial and it is unclear whether the value of interest is the 4-week score or the change from baseline to 4-weeks | Not applicable | Not defined: unclear if the value for each individual is continuous or categorical. | Baseline, 4-weeks |
| *Complete* | | | | | |
| Change from baseline in HAM-D17 score at 4-weeks (continuous outcome) | 17-item Hamilton Depression Rating Scale (HAM-D17) | Change from baseline | Not applicable | Continuous | Baseline, 4-weeks |
| Mean change from baseline in HAM-D17 [Baseline, 4-weeks] | 17-item Hamilton Depression Rating Scale (HAM-D17) | Change from baseline | Not applicable | Continuous | Baseline, 4-weeks |

| Example 3 | Specific measurement | Specific metric | Cutoff | Variable type | Time point |
| --- | --- | --- | --- | --- | --- |
| *Incomplete* | | | | | |
| Change in HbA1c during the intervention period compared to the control period [Baseline, 8-weeks, 12-weeks, 16-weeks] | HbA1c | Not defined: (1) unclear if “change” refers to within-participant change or the between-group change, (2) 4 time points are reported, unclear if the metric of interest is change between two values (e.g., change from baseline) or rate of change over time | Not applicable | Not defined: “change in” is usually a continuous score, but it can also be categorical (change or not) | Not defined: unclear if the four time points are for one outcome or multiple outcomes |
| *Complete* | | | | | |
| Change in HbA1c from baseline at 8-weeks (continuous outcome) | HbA1c | Change from baseline | Not applicable | Continuous | Baseline, 8-weeks |
| HbA1c slope of change from baseline, estimated across participants by mixed-effects model [Baseline, 8-weeks, 12-weeks, 16-weeks] | HbA1c | Set of values at multiple time points | Not applicable | Continuous | Baseline, 8-weeks, 12-weeks, 16-weeks |

| Example 4 | Specific measurement | Specific metric | Cutoff | Variable type | Time point |
| --- | --- | --- | --- | --- | --- |
| *Incomplete* | | | | | |
| Major Adverse Cardiovascular Events (MACE) [12-months] | Not defined: MACE is a composite outcome, unclear which specific events would be included in the definition | Not defined: unclear whether the variable of interest is the binary status of each participant experiencing MACE or the time to the first occurrence of MACE. | Not applicable | Not defined: unclear whether the variable of interest is the binary status of each participant experiencing MACE or the time to the first occurrence of MACE. | 12-months |
| *Complete* | | | | | |
| Whether a patient experienced Major Adverse Cardiovascular Event (MACE). MACE is defined as having one of the following within the 12-month follow-up period: cardiovascular death ascertained from death certificates, nonfatal MI diagnosed by troponin elevation and ECG/imaging changes, or nonfatal stroke diagnosed by neurologic deficit and imaging confirmation. | MACE, defined as:  cardiovascular death ascertained from death certificates  nonfatal MI diagnosed by troponin elevation and ECG/imaging changes, or  nonfatal stroke diagnosed by neurologic deficit and imaging confirmation | Value at (or occurrence by) a time point | Not applicable | Categorical | 12-months |
| Median time to first occurrence of Major Adverse Cardiovascular Events (MACE). MACE is defined as the occurrence of CV death, non-fatal myocardial infarction (MI), or non-fatal stroke. [12 months] | MACE, defined as:  cardiovascular death ascertained from death certificates  nonfatal MI diagnosed by troponin elevation and ECG/imaging changes, or  nonfatal stroke diagnosed by neurologic deficit and imaging confirmation | Time to event | Not applicable | Continuous | 12-months |
