# Supplementary material for: Guidance for defining outcomes in clinical trials
Source: PLoS Med. 2026 Oct 21;23(10):e1005275. doi: 10.1371/journal.pmed.1005275 (PMC13719018; doi:10.1371/journal.pmed.1005275)
Supplement: Supplementary file 3 [file pmed.1005275.s004.docx]

## S1 Table. Frameworks for defining outcomes

| Element | Zarin 2011 | SPIRIT and CONSORT† | Current six-element framework |
| --- | --- | --- | --- |
| Outcome domain | No definition provided, example given (“anxiety”) | “Title or concept to describe≥1 outcomes” | The concept (e.g., depression) or phenomenon (e.g., death) being assessed. |
| Specific measurement | No definition provided, example given (“Hamilton Anxiety Rating Scale”) | “Corresponds to the data collected directly from trial participants; description includes the instrument used to assess the outcome domain - Descriptive name - If applicable, the total score or the subscales that will be analyzed” | The name of a scale or instrument used to assess a concept, or the name of a phenomenon that can be observed directly. |
| Specific metric | “to characterize each participant's results (e.g., change from baseline, end value, time to event)” | “Participant-level unit of measurement (eg, change from baseline, final value or a value at a time point, time to event) for analysis” | How the participant-level quantity or quantities of interest are obtained from a participant's assessments at one or more time points. |
| Method of aggregation | “method of aggregating data within each group (e.g., a categorical measure such as proportion of participants with a decrease greater than or equal to 50%)” | “The procedure for estimating the treatment effect - If the outcome will be treated as a continuous, categorical, or time-to-event variable - For continuous variables, a measure of central tendency (eg, mean value); for categorical and time-to-event data variables, proportion with an event and, if relevant, the specific cutoff values or categories compared” | Replaced with two elements:  1. Cutoff  The specific numeric cutoff(s) or category(ies) used to define the value for each individual. When applicable, a cutoff can be described using three sub-elements:   a. Value being categorized (i.e., specific measurement and specific metric); b. Threshold or categories imposed on the individual values; c. Direction of interest or operational signs.   2. Variable type  Whether the value for each individual is treated as continuous or categorical. |
| Time point | “time frame” for each outcome | “The timing of follow-up measurements”  “When outcome measurements will be obtained”  “Which of the outcome measurements will be analyzed” | A moment when an assessment will be recorded. |

†Include SPIRIT and CONSORT-Outcomes extensions 2022 and SPIRIT and CONSORT 2025; all quotations from SPIRIT and CONSORT-Outcomes extensions 2022
