# Supplementary material for: Guidance for defining outcomes in clinical trials
Source: PLoS Med. 2026 Oct 21;23(10):e1005275. doi: 10.1371/journal.pmed.1005275 (PMC13719018; doi:10.1371/journal.pmed.1005275)
Supplement: Supplementary file 4 [file pmed.1005275.s005.docx]

## S2 Table. Common metrics describing values for individual participants in clinical trials^*^†

| Metric | Definition | Example |
| --- | --- | --- |
| Value at (or occurrence by) a time point | A single value characterizing the participant with respect to one specified time, which might be a value measured at that time or the occurrence of an event at or before that time.  Multiple assessments might be needed to ascertain this value, but the quantity itself is defined at one moment. | Insomnia Severity Index - total score at 8 weeks (continuous outcome) [NCT01987089]    Whether or not the infant was readmitted to the hospital within 6 weeks postpartum (binary) [NCT04296396] |
| Change between two time points | Difference in value between two time points (e.g., change between baseline and follow-up). Might be an absolute or relative difference on a continuous or ordinal scale, or a shift between categories.    Not applicable if the outcome is assessed once, even if the assessment focuses on change (e.g., Patient Global Impression of Change Scale assessed only at the end of treatment). | Change from baseline in lumbar spine bone mineral density (BMD) Z-score at month 12 (continuous outcome) [NCT00799266]  Change in New York Heart Association (NYHA) Classification from baseline to week 4 (categorical variable) [NCT02468232]  Change of Sequential Organ Failure Assessment (SOFA) score between day 1 and day 7 (continuous outcome) [NCT04330638] |
| Set of values at multiple time points | The values at multiple assessment time points, entering the analysis as they are rather than combined into a single summary per participant. | Geographic atrophy area measured by fundus autofluorescence at Baseline, Month 6, and Month 12, with the rate of change estimated across participants (continuous outcome) [NCT04435366] |
| Slope per participant | “Slope” of change across three or more time points per participant. | The individual participant’s annualized change (slope) of eGFR, fitted separately for each patient from their eGFR assessments over 24 months (i.e., at baseline and every 3 months) (continuous outcome) [NCT02795676] |
| Variability per participant | Variability of more than two continuous values per participant (e.g., SD, range, IQR). | Standard deviation of all the blood glucose values measured during the first 48 hours following the intervention (assessment at Baseline, immediately after transcatheter aortic valve implantation, 24 hours, and 48 hours) (continuous outcome) [NCT02726958]    Ambulatory systolic blood pressure variability (measured by coefficient of variation) within the first 6 weeks of treatment (intermittent assessment every 15–20 minutes during the day and every 30 minutes at night over 24 hours) (continuous outcome) [NCT04931108] |
| Average per participant | Average of two or more continuous values per participant, or average of events or discrete occurrences per participant (i.e., sum divided by the number of assessments or time for that individual). | Average of daily longest sleep duration recorded each day over the 7-day sleep log period (continuous outcome) [NCT04864249]  Mean Amplitude of Glycemic Excursions (MAGE) per participant during hospitalization (up to 10 days) (continuous outcome) [NCT03877068]  Average number of daily interactions with the device as recorded by the device within 90 days post enrollment (continuous outcome) [NCT04394754] |
| Sum per participant | Total amount or weighted total of a continuous measure (e.g., biomarker) over time.  Sum of events or discrete occurrences per participant (i.e., total number of events or occurrences observed during the study period). | Total area under the curve from time zero to extrapolated infinite time (AUCinf) of factor VIII activity (continuous outcome) [Time Frame: 0.5, 8, 24, 28 and 32 hours post dose on Day 1] [NCT00543439]    Cumulative oral corticosteroid dose over the treatment period (52 weeks) (continuous outcome) [NCT02383589]    Number of visits with platelet counts ≥50x10^9/L over the Treatment Period of the study (Week 1 to Week 25) (continuous outcome) [NCT04200456] |
| Maximum per participant | Maximum value of continuous values per participant over time. | Maximum observed plasma concentration of ibrutinib, sample collected at 1, 2, and 4 hours after administration of ibrutinib (continuous outcome) [NCT01776840]  Peak gametocyte density measured by qRT-PCR over multiple assessment windows within 51 days after challenge infection (continuous outcome) [NCT03454048] |
| Minimum per participant | Minimum value of continuous values per participant over time. | Minimum observed plasma concentration of ibrutinib, sample collected at 1, 2, and 4 hours after administration of ibrutinib (continuous outcome) [NCT01776840]  Nadir Systolic Blood Pressure during hemodialysis in mmHg, Systolic Blood Pressure is measured every 15 minutes during each study hemodialysis treatment (up to 36 treatments per participant over 15 weeks) (continuous outcome) [NCT03301740] |
| Time-to-event | Time to the occurrence of an event. | Kaplan-Meier recurrence-free survival probability for each limb affected by clubfoot up to 1 year after bracing is discontinued. Recurrence is defined as the development of any of the following deformities in isolation or in combination that require repeat cast application or surgical intervention: hindfoot varus, ankle equinus, midfoot adduction, midfoot cavus, or forefoot pronation. Clubfoot recurrence was determined by the local Principal Investigator [NCT01551264]  Time to recovery of testosterone to > 230 ng/dL during the first five years of follow-up (continuous outcome) [NCT01546987] |
| Duration | Length of time during which an event occurs, or proportion (or percentage) of time during which an event occurs. | Length of hospital stay while on study (continuous outcome) [Time Frame: from randomization up to 1 year following birth] [NCT01029353]  Percentage of time with sensor glucose values between 70 and 180 milligrams per deciliter (mg/dL), efficacy estimand at week 16 (continuous outcome) [NCT03830281] |

**^*^**Some outcomes incorporate multiple specific metrics to arrive at a final score for each individual. For example, the outcome “Change in Mean Amplitude of Glycemic Excursions (MAGE) from baseline” involves two metrics: "Average per participant" and "Change between two time points." That is, each individual participant’s score is calculated by first taking the arithmetic mean of the amplitudes of glycemic excursions (peak to nadir or nadir to peak) that exceed one standard deviation of the glucose values and second by calculating the difference between the MAGE at follow-up and the MAGE at baseline.

†Examples are adapted from outcomes registered on ClinicalTrials.gov (NCT numbers in brackets). Wording has been edited for clarity and consistency with the framework and might differ from the registry record.
