# Supplementary material for: Guidance for defining outcomes in clinical trials
Source: PLoS Med. 2026 Oct 21;23(10):e1005275. doi: 10.1371/journal.pmed.1005275 (PMC13719018; doi:10.1371/journal.pmed.1005275)
Supplement: Supplementary file 5 [file pmed.1005275.s006.docx]

## S3 Table. Do's and don'ts when defining time points (adapted from ClinicalTrials.gov)

| **Do** | **Don’t** |
| --- | --- |
| State an upper bound or bounded period: e.g., “up to 24-weeks”;  When a vague phrase is unavoidable, include an estimated duration: e.g., “through study completion, an average of 1 year” | State open-ended timeline only: e.g., “through study completion”; “until end of study”  State frequency only: e.g., “Every 3 weeks” |
| Anchor to the participant's own timeline: e.g., “from enrollment to end of treatment at 8-weeks” | Use trial’s administrative timeline:  e.g., “until end of study”  Use calendar dates:  e.g., “2012-2013”; “January 2020” |
| Use a standard unit of time: e.g., “Day 1,” “Month 3” | Use one-letter abbreviations: e.g., “D1,” “M3” |
| When assessment is tied to an intervention or event, define by that event (a numeric time point isn't required): e.g., intraoperative, perioperative, preoperative, immediately before/after, pre-dose, during procedure | State open-ended event-based timeline: e.g., “postoperative” |
| For outcomes derived from multiple assessments, list every contributing time point or state the assessment schedule:   - Time-to-event outcomes: state origin, maximum follow-up, and assessment frequency (e.g., “from randomization up to 100-months, assessed every 6-months”) - Pharmacokinetic (Cmax, AUC): list the multiple collection time points/intervals (e.g., “1, 2, 3, 4, 5, 6, 8, 12, 24, 48, 72, and 96 hours post-dose”)   Measures of change between two points or over time: specify two or more time points (e.g., “at baseline and at 6-months”) | List multiple timepoints together when assessment at each is an individual outcome: e.g., “Oswestry Disability Index (ODI) at Baseline and at 2-year”, “Glycated Hemoglobin (HbA1c) at Baseline, 3-, 6-, 9-months” |
