# Supplementary material for: Guidance for defining outcomes in clinical trials
Source: PLoS Med. 2026 Oct 21;23(10):e1005275. doi: 10.1371/journal.pmed.1005275 (PMC13719018; doi:10.1371/journal.pmed.1005275)
Supplement: Supplementary file 6 [file pmed.1005275.s007.docx]

## S4 Table. Outcome fields of 18 WHO recognized primary registers*

| **Registry** | **Outcome fields** | **Designated field for specific measurement** | **Designated field for time point** | **Publicly available field explanations** |
| --- | --- | --- | --- | --- |
| Australian New Zealand Clinical Trials Registry (ACTRN) | Primary outcome  Assessment methods  Timepoint(s)  Secondary outcome  Assessment methods  Timepoint(s) | **✔** | ✔ | https://www.anzctr.org.au/docs/ANZCTR%20Data%20field%20explanation.pdf?t=569 |
| Brazilian Clinical Trials Registry (ReBec) | Primary outcomes  Secondary outcomes |  |  |  |
| Chinese Clinical Trial Registry (ChiCTR) | Outcome  Type  Measure timepoint of outcome  Measure method | **✔** | ✔ |  |
| Clinical Research Information Service (CRiS), Republic of Korea | Type of primary outcome  Primary outcome(s)   - Outcome - Timepoint   Secondary outcome(s)   - Outcome - Timepoint |  | ✔ |  |
| Clinical Trials Information System (CTIS) | Primary end points  Secondary end points |  |  |  |
| Clinical Trials Registry India (CTRI) | Primary outcome  Outcome  Timepoints  Secondary outcome  Outcome  Timepoints |  | ✔ | https://ctri.nic.in/Clinicaltrials/CTRI_Dataset_and_Description.pdf |
| Cuban Public Registry of Clinical Trials (RPCEC) | Primary outcome(s)  Key secondary outcomes |  |  | Instructions embedded within each registration |
| European Union Drug Regulating Authorities Clinical Trials Database (EudraCT) | Primary end point(s)  Timepoint(s) of evaluation of this end point  Secondary end point(s)  Timepoint(s) of evaluation of this end point |  | ✔ |  |
| Deutsches Register Klinischer Studien (DRKS) [German] | Login required for accessing details of registrations at the time of our study |  |  |  |
| Iranian Registry of Clinical Trials (IRCT) | Inaccessible at the time of our study |  |  |  |
| International Standard Randomised Controlled Trial Number (ISRCTN) | Primary outcome measure(s)  Key secondary outcome measure(s) |  |  | <https://www.isrctn.com/page/definitions#primary-outcomes-who> |
| International Traditional Medicine Clinical Trial Registry (ITMCTR) | Outcome name  Type  Measure timepoint of outcome  Measure method | **✔** | ✔ |  |
| Japan Registry of Clinical Trials (jRCT) | Primary Outcome(s)  Secondary Outcome(s) |  |  |  |
| Lebanese Clinical Trials Registry (LBCTR) | Primary outcomes   - Name - Time points - Measure   Key secondary outcomes   - Name - Time points - Measure | **✔** | ✔ | https://lbctr.moph.gov.lb/Files/User%20Guide.pdf |
| Thai Clinical Trials Registry (TCTR) | *P*rimary Outcome   - Outcome Name - Metric / Method of measurement - Time point   Secondary Outcome   - Outcome Name - Metric / Method of measurement - Time point | **✔** | ✔ |  |
| Pan African Clinical Trial Registry (PACTR) | Type of outcome  Outcome  Timepoint(s) at which outcome measured |  | ✔ |  |
| Peruvian Clinical Trial Registry (REPEC) | Inaccessible at the time of our study |  |  |  |
| Sri Lanka Clinical Trials Registry (SLCTR) | Primary outcome(s)  Secondary outcome(s) |  | ✔ |  |

*Trial registers do not include structured fields for defining each element of an outcome, and only a few registers provide guidance concerning what information should be included in these fields to ensure outcomes are defined completely.
